# Changes in global DNA methylation under climatic stress in two related grasses suggest a possible role of epigenetics in the ecological success of polyploids

**DOI:** 10.1038/s41598-022-12125-4

**Published:** 2022-05-18

**Authors:** Przemysław P. Tomczyk, Marcin Kiedrzyński, Ewa Forma, Katarzyna M. Zielińska, Edyta Kiedrzyńska

**Affiliations:** 1 Department of Biogeography, Paleoecology and Nature Conservation, Faculty of Biology and Environmental Protection, University of Lodz, Banacha 1/3, 90-237 Lodz, Poland; 2 The National Institute of Horticultural Research, Konstytucji 3 Maja 1/3, 96-100, Skierniewice, Poland; 3 Department of Cytobiochemistry, Faculty of Biology and Environmental Protection, University of Lodz, Pomorska 141/143, 90-236 Lodz, Poland; 4 European Regional Centre for Ecohydrology of the Polish Academy of Sciences, Tylna 3, 90-364 Lodz, Poland; 5 UNESCO Chair On Ecohydrology and Applied Ecology, Faculty of Biology and Environmental Protection, University of Lodz, Banacha 12/16, 90-237 Lodz, Poland

**Keywords:** Biogeography, Climate-change ecology, Molecular ecology, Ecology, Environmental sciences, Plant sciences, Light responses, Natural variation in plants, Plant ecology, Plant molecular biology, Plant stress responses

## Abstract

Polyploidization drives the evolution of grasses and can result in epigenetic changes, which may have a role in the creation of new evolutionary lineages and ecological speciation. As such changes may be inherited, they can also influence adaptation to the environment. Populations from different regions and climates may also differ epigenetically; however, this phenomenon is poorly understood. The present study analyzes the effect of climatic stress on global DNA methylation based on a garden collection of two related mountain grasses (the narrow endemic diploid *Festuca tatrae* and the more widely distributed mixed-ploidy *F*. *amethystina*) with different geographic ranges and ecological niches. A lower level of DNA methylation was observed for *F. tatrae*, while a higher mean level was obtained for the diploid and tetraploid of *F. amethystina*; with the tetraploids having a higher level of global methylated DNA than the diploids. The weather conditions (especially insolation) measured 24 h prior to sampling appeared to have a closer relationship with global DNA methylation level than those observed seven days before sampling. Our findings suggest that the level of methylation during stress conditions (drought, high temperature and high insolation) may be significantly influenced by the ploidy level and bioclimatic provenance of specimens; however an important role may also be played by the intensity of stress conditions in a given year.

## Introduction

The global environment is undergoing rapid transformation, with climate change playing a considerable role^1^. To survive in these changing conditions, plants can use their plasticity, undergo adaptation or track the suitable conditions by range shift; if this is not possible, they will most likely become extinct^2^. Climate change also has a considerable impact on the production potential of agriculture, by changing the production potential^3,4^. To mitigate the effects of climate change, these is hence a pressing need to protect ecosystem diversity and stability, two challenges that require greater knowledge of plant adaptation^5^ and to identify the traits and mechanisms that drive plant adaptation to environmental change and identify those that can mitigate the effects of climate change.

One promising direction in research into plant adaptation is based around the study of epigenetics^4^, i.e. changes to the genome architecture, often functionally relevant, that do not involve a change in the nucleotide sequence itself. Such changes may be (but not always) meiotically or mitotically-heritable^6^. At the molecular level, epigenetic phenomena are mediated by reversible marks such as DNA methylation, histone modifications, small RNAs (sRNAs) and in some cases, by microRNAs (miRNAs) that can alter the regulatory states of genes or genomic regions^6^. Epigenetic processes may have a significant influence on the response to abiotic stress and other environmental challenges, and the plant epigenome is known to respond swiftly to environmental cues and developmental changes^7^.

The present study uses DNA methylation, one of the most widely-studied epigenetic modifications^6^. The DNA methylation process, believed to protect the genome against unfavorable biotic and abiotic stresses, can be rapidly and dynamically affected by environmental changes^7–9^. There is growing evidence that plant DNA methylation is affected by climatic factors; for example, temperatures lower than norms generally decrease the global level of DNA methylation^10^, while salt stress increases it^11^. In addition, DNA methylation also often increases in response to drought, as documented for *Pisum sativum*^12^ or *Oryza sativa*^13^.

An interesting model for research into the epigenetic adaptability of plants to stress conditions is based on the use of two closely related mountain grasses (Poaceae): *Festuca amethystina* L. and *Festuca tatrae* (Czakó) Degen^14,15^. Those fine-leaved fescues differ significantly in distribution: *F. amethystina* is widely distributed across the Central European mountains with some lowland localities, while *F. tatrae* is strictly mountain species—endemic for the Western Carpathians^16,17^. Moreover, *Festuca amethystina* is a mixed-ploidy species, with diploid and tetraploid cytotypes^18^; of these, the tetraploid forms tend to be observed at lower altitudes, and across a wider range of climates, habitats and geology^19^. *F. tatrae* is only known to exist in diploid forms and in narrow habitat conditions^14^.

We hypothesize that the ploidy level differentiation within *F. amethystina* improves its chance of evolutionary and ecological success by extending its adaptability. In fact, many papers report that phenotypic plasticity in newly-formed polyploids promotes niche expansion, and that polyploid taxa have greater tolerance to stressful conditions than their parental species^20^. The present study explores this hypothesis, examining the differences in global DNA methylation in a common garden experiment, as recommended in epigenetic studies^21^.

As the plant epigenome responds to environmental conditions, and the resulting changes may be inherited, populations from different regions and environments may differ epigenetically^6^. Therefore, the present study uses a living collection of specimens of *F. amethystina* and *F. tatrae* taken from different localities distributed across the whole area of their species ranges.

Our study compares the global DNA methylation profiles formed under stress conditions with that observed under more optimal conditions and addresses the following research questions:

1. What changes in global DNA methylation are observed in specimens of *F. amethystina* and *F. tatrae* kept in common garden conditions, grown under stress (drought, high temperature and high insolation) and under more favorable conditions, according to species and ploidy level?
2. Which climatic factors, soil humidity or phenotypic characteristics of specimens measured directly during the experiment are most closely related to DNA methylation level?
3. Which characteristics of the original provenance of the plants are most closely related to the DNA methylation levels in a common garden conditions?

## Results

### Global DNA methylation in stress and control conditions

**Figure 1:**
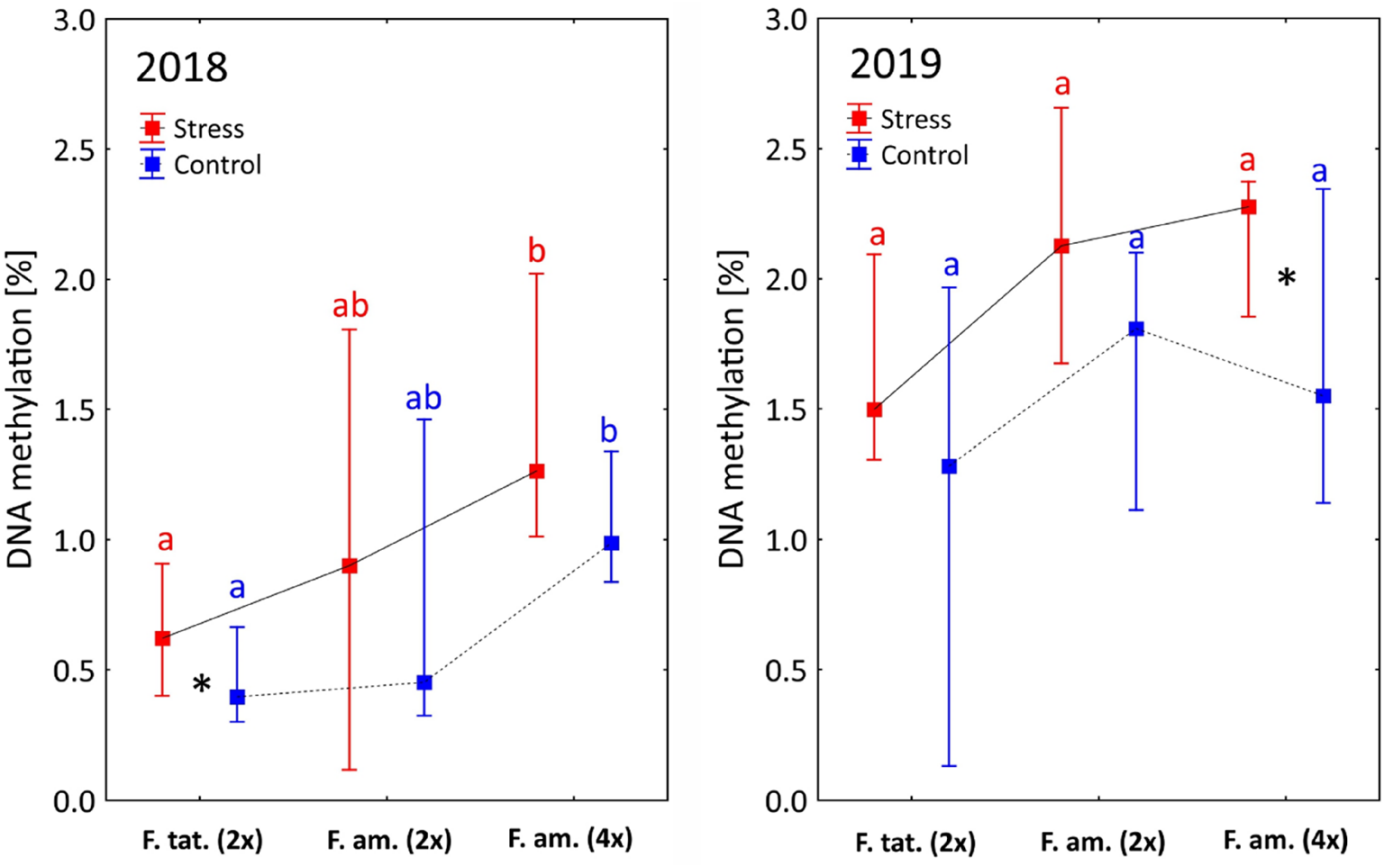
Global DNA methylation level for species (and cytotypes) in a given year and sampling conditions (stress vs. control). Squares—median values; whiskers—upper and lower quartile. Letters denote statistical significance (< 0.05) of DNA methylation level between species/cytotypes in the same year and the same sampling conditions, according to the non-parametric Kruskal–Wallis test. An asterisk indicates significant (p < 0.05) differences in DNA methylation levels between stress and control conditions within the species/cytotype in the same year according to the Wilcoxon pairwise test.

A higher level of median DNA methylation was observed in all groups (species and cytotypes) during climatic stress (i.e. thermal, drought and insolation) (Fig. 1). Higher levels of DNA methylation were observed in 2019: the median values did not exceed 1.5% in 2018; however, they did not fall below 1.2% the next year (Fig. 1).

Of the two studied species, *F. amethystina* demonstrated higher mean global DNA methylation values in both 2018 and 2019, with the highest values being achieved by the tetraploid *F. amethystina*. In 2018, statistically significant differences were only observed between *F. tatrae* and tetraploids of *F. amethystina* (Fig. 1); in 2019, only slight differences were observed between cytotypes.

**Figure 2:**
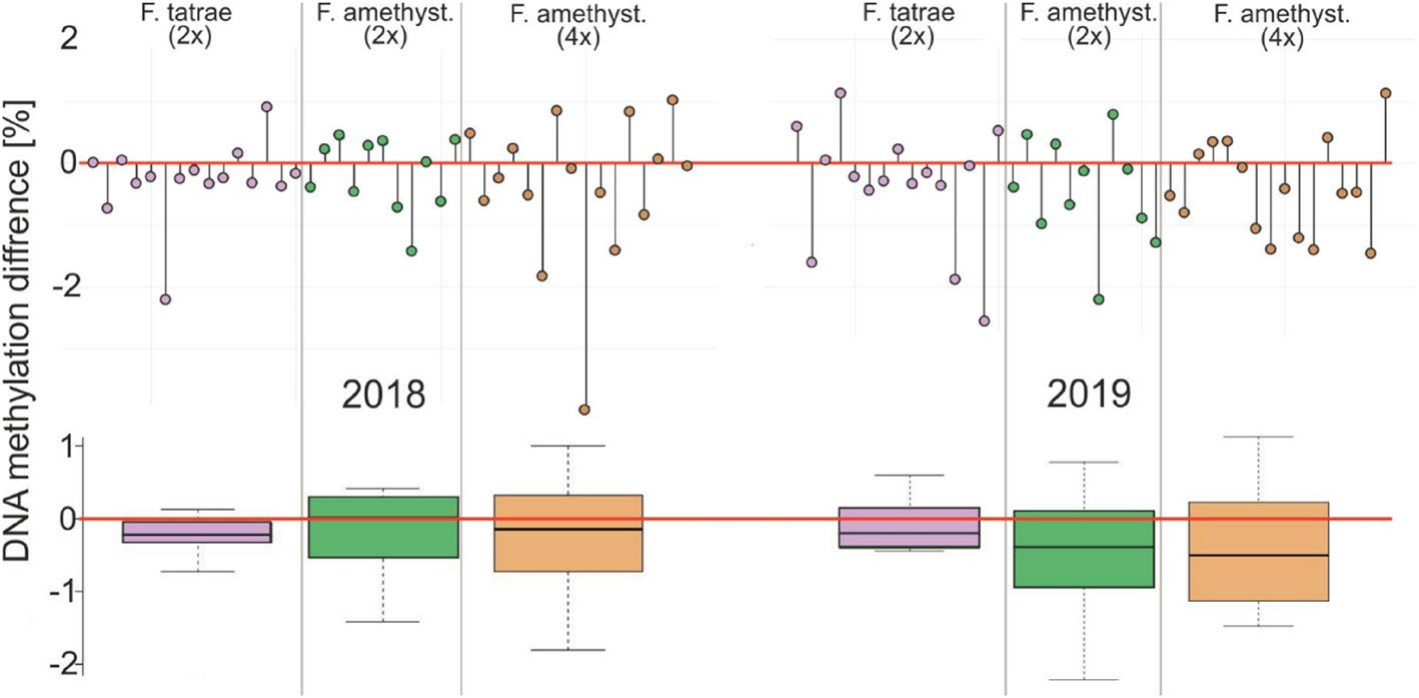
Variation in the global DNA methylation level between stress and control conditions in the common garden experiment in a given year, for studied specimens (point plots) and for species and cytotypes (boxplots: horizontal line—median, boxes—25–75th percentile, whiskers—non-outliers).

The variability of the epigenetic reaction of individual plants, i.e. the difference in the degree of DNA methylation between stress and control conditions, differed between species and cytotypes (Fig. 2). In both years, the greatest decrease in DNA methylation from stress to control conditions was noted for the tetraploid *F. amethystina* (Fig. 2). A smaller decrease was observed for the diploids of both species; however, no differences in median DNA methylation were demonstrated in diploid *F. amethystina* in 2018. Both diploid and tetraploid specimens of *F. amethystina* demonstrated visibly greater changes in DNA methylation between stress and control conditions in 2019 compared to 2018 (Fig. 2).

For all individuals, significant (p < 0.05) but moderate positive correlations in global DNA methylation levels were found between stress and control conditions within each measurement year (Pearson’s coefficient: 0.34 for 2018 and 0.40 for 2019), i.e. individuals with a higher level of DNA methylation under stress conditions also maintained a higher level of methylation under control conditions. Consequently, the differences in methylation in a given year between stress and control were negatively correlated with the level of methylation during stress (− 0.85 for 2018 and − 0.46 for 2019): those specimens with higher methylation during stress demonstrated smaller differences.

**Figure 3:**
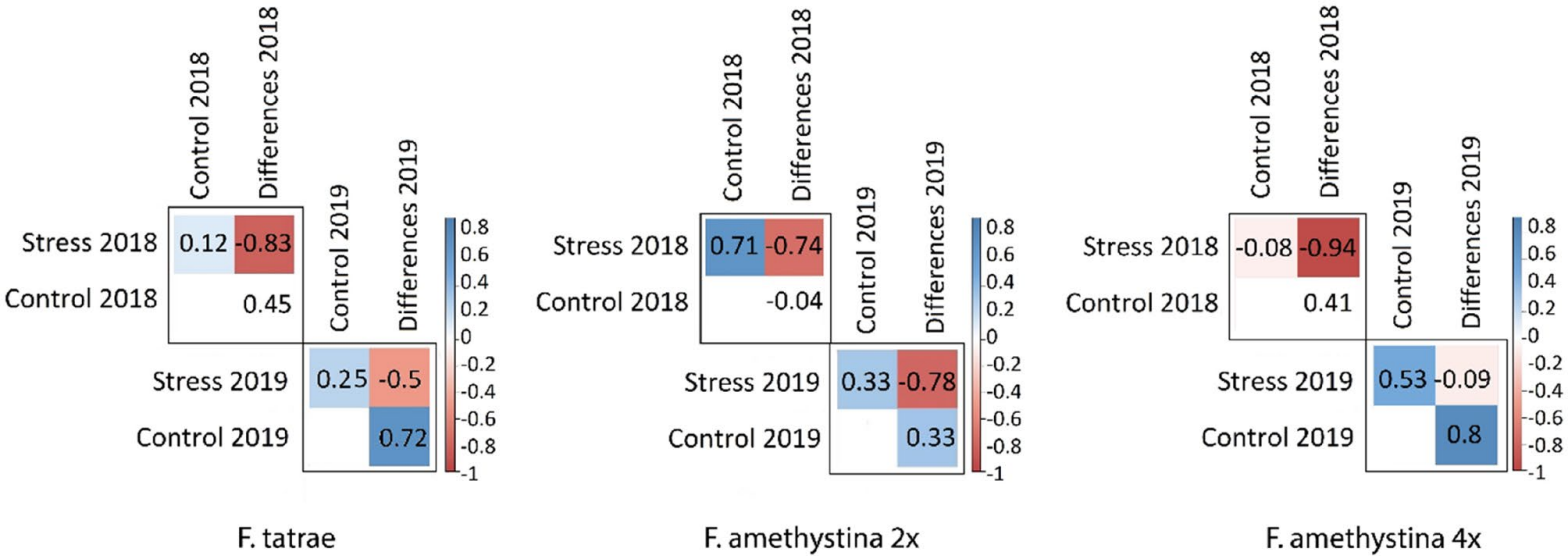
Correlation matrices for the obtained levels of global DNA methylation during stress and control conditions—calculated separately for each species and cytotypes: *F. tatrae*, *F. amethystina* 2 × and *F. amethystina* 4 ×. Blue and red fields—statistically significant negative and positive correlations respectively; white fields have statistically insignificant correlations (p < 0.05).

Further correlations between DNA methylation levels were calculated separately for each species and cytotype; the results indicate that this adverse relationship between stress and controls was only repeated for diploids of *F. amethystina* (Fig. 3). However, *F. tatrae* demonstrated no correlation (0.12) between stress and control conditions in 2018. In addition, tetraploid *F. amethystina* demonstrated no correlation in methylation level between stress and control in 2018 (− 0.08), nor between stress and controls in 2019 (− 0.09) (Fig. 3).

### Factors affecting the level of global DNA methylation

The presence of adverse weather conditions, such as higher insolation and lower air humidity, measured 24 h and not seven days before sampling was associated with an elevated level of DNA methylation (as noted in 2019). In both years, higher temperature and humidity deficiency, indicative of stress conditions, were associated with a relatively higher level of DNA methylation compared to the control periods in the same year (Supplementary Fig. S1).

**Figure 4:**
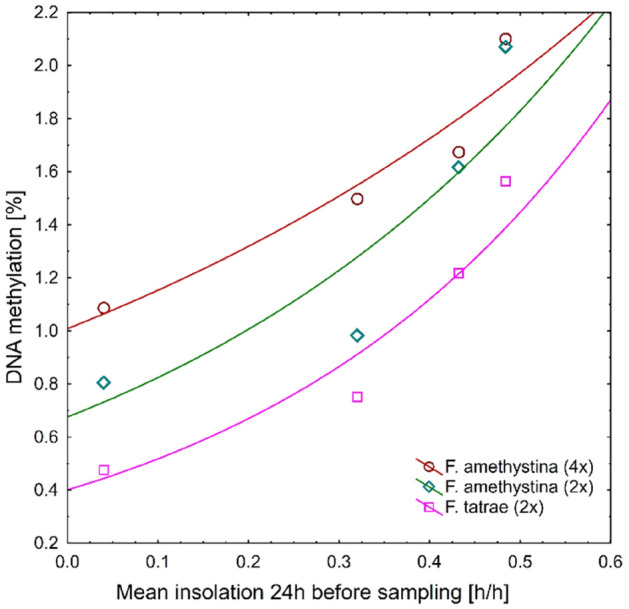
The exponential relationship between mean global DNA methylation level and mean insolation 24 h before material sampling, according to species and ploidy level, in the common garden experiment with *F. amethystina* and *F. tatrae*. Global DNA methylation values are means for all studied specimens in each group and at each sampling date.

For both studied species and all cytotypes, an exponential relationship was observed between DNA methylation level and mean insolation (in hours per hour) 24 h before sampling (Fig. 4).

For three of the four analyzed sampling dates, bioclimatic parameters describing the environmental provenance of the individuals were found to be more important for explaining global DNA methylation level than those recorded during the experiment (i.e. phenotypic features of the plants and soil moisture) (Table 1). The temperature variables were found to give a better explanation of the methylation level than those related to precipitation, and this is particularly clear in the final Model set 3 (Table 1). It is worth noting that one of the most significant variables was the mean diurnal range of temperature (Bio2) in the original locations from where the plants were obtained repeats in models (Table 1).

**Table 1:** The most significant factors influencing global methylation level in the common garden experiment with *F. amethystina* and *F. tatrae* accordingly to stepwise regression. Calculations were performed separately for three types of data sets, detailed results are included in Supplementary Tables S2–S5. Significance codes: ***< 0.001; **0.001; *0.01; none > 0.05.

Analysed conditions	Model set 1 (soil moisture + phenotypic data + chromosome number)	Model set 2 (altitude + bioclimatic variables of original location)	Model set 3 (the most significant variables from Model set 1 and Model set 2)
Stress 2018	Chromosome number*	Bio2*	Bio2*
		Bio9*	Bio9*
Control 2018	Chromosome number**	Bio12**	Chromosome number*^,^** (*significance depends on the model, the smallest importance it has combined with bio12*)
		Bio13**	
		Bio14**	
		Bio16**	
		Bio17**	
		Bio19**	
Stress 2019	Soil moisture	Bio7***	Bio8***
		Bio8***	Bio7**^,^***
		Bio5**	Bio4*^,^**
		Bio6**	Bio5**
		Bio4*,**	Bio6*
		Bio2*^,^**	Bio2*
Control 2019	*No significant variables*	Bio2*	Bio2

Our results indicate that the level of DNA methylation during stress may be significantly influenced by the climatic provenience of the specimens. The climate parameters from the original location, such as temperature extremes and the range between them, as well as the temperatures characterizing the driest or wettest parts of the year, had the greatest influence. In 2018, Bio2 and mean temperature of driest quarter (Bio9) were most important for explaining DNA methylation level in stress conditions; in 2019, Bio2 was supplemented by a further five variables in stress conditions (Table 1).

Ploidy level was found to be the main factor for explaining methylation level during control conditions in 2018, and Bio 2 for control conditions in 2019. Taken as a whole, the data indicates that the results obtained for the control period in 2018 clearly differ from those of the other periods (Table 1).

## Discussion

In our experiment, a higher methylation level was generally observed during stress conditions than in unstressed controls (Fig. 1), and this is in line with general knowledge on plant epigenetic reactions (cf.^22^). However, even closely-related species can demonstrate considerable variations in DNA methylation^23^ and hence may respond differently to changing environmental conditions^24^. In our case*, F. amethystina* demonstrated a generally higher mean level of global DNA methylation than its close relative *F. tatrae.* Although *F. amethystina* has mixed levels of ploidy, with the tetraploid form having generally higher methylation levels, even the diploid *F. amethystina* demonstrated higher DNA methylation levels than the diploid *F. tatrae*, despite having a similar genome size. However, the present study tested global DNA methylation, without any distinction between CG, CHG and CHH methylation; as such, any conclusions drawn on the differences between the studied species need to be confirmed in further experiments. Furthermore, methylation level can be influenced by numerous other phenomena, such as genome shuffling^25^, genomic shock^26^ and subsequent repatterning of expression^27^.

It would be easy to attribute the higher level of methylation in tetraploid plants to its larger genome, as in Róis et al.^28^, but research on cytotypes within species indicate that usually no such clear relationship exists (e.g.^29^). Similar results were also obtained in the present study, with any such changes being dependent on the studied season. Models obtained through step regression (Table 1) indicate that ploidy level plays a key role in explaining the methylation level observed during control conditions in 2018. In 2019, during control conditions, the tetraploid *F. amethystina* demonstrated lower DNA methylation levels than the diploids (Fig. 1), and the diploids demonstrated weaker vitality than recorded in previous years: most of them were not flowering. These findings suggest that the higher level of DNA methylation observed in diploids may be influenced by the general condition of the plants and their development, and not by ploidy level per se.

It has been reported that DNA methylation patterns may differ between varieties of the same species^30,31^. In the present study, the responses differed between *F. amethystina* plants and cytotypes, as well as between only diploid *F. tatrae* (Fig. 2). Similarly, Zheng et al., found varieties of rice to respond to stress in different ways at the DNA methylation level^32^; these findings were supported by those from further studies on rice indicating that different genotypes, and even tissues, demonstrate differences in cytosine methylation under salinity stress, irrespective of the level of salinity tolerance demonstrated by the genotype^33^.

Our present findings also indicate that, in most cases, plants characterized by a higher methylation level during stress demonstrated smaller differences between stressful and conducive conditions. An exception to this rule was the reaction demonstrated by tetraploids of *F. amethystina* in 2019, when any significant change in methylation caused by stress was found to return under normal conditions. It could be interpreted that higher methylation levels may cause slower demethylation processes during the transition to non-stress conditions. Wang et al*.*^13^ and Yaish^8^ found that in rice subjected to increasing DNA methylation during drought stress, only 70% of the total changes reset to the normal level after returning to non-drought conditions.

The idiosyncratic and variable behavior of the tetraploids of *F. amethystina* could be explained by the complex structure of the genome, driven in this case by a probable allopolyploid origin^34^. In the case of polyploidy, more suitable DNA methylation modifications can reduce the degree of incompatibility that arises from the presence of two or more genomes in a nucleolus^27^, it also takes part in transposon silencing. However, previous studies of hybrid plants found that while changes in their DNA sequence appeared to be more, or less, additive compared to the parental species, genome methylation and gene expression were not^35^. Another possible explanation for the variable DNA methylation responses displayed by tetraploids of *F. amethystina* could derive from their wider biogeographical niche and the higher number of original habitats in which they occur; this could potentially drive different adaptations^19^. Hence, further research based on genomic data is required to fully understand the relationship between allopolyploid plasticity and methylation levels.

In plants, DNA methylation seems to be very dynamic and changeable^24^. In our experiment, weather conditions taken 24 h before sampling explain global DNA methylation values better than those taken seven days before sampling. Studies have indicated a change in DNA methylation as a reaction to external factors after seven days of exposure^36^ or even after 1 h^37^. In the present study, a particularly strong relationship was observed between DNA methylation level and insolation 24 h before sampling (Fig. 4). This is probably caused by the effect of UV radiation, which is harmful to living organisms, the increase in cell transpiration through the leaves, and the more noticeable drought. Recent research has shown that insolation is a key driver of short-term changes in DNA methylation, and thus the stress experienced by the plant, as well as their physiological changes and adaptation to harmful conditions^38,39^. However, it seems that there is no universal rule governing the DNA methylation response of plants to UV radiation: UV-B radiation elicited DNA demethylation in *Artemisia annua*^40^ and UV-A/B irradiation resulted in minimal changes in DNA methylation in maize^41^. However, the strong and exponential relationship between insolation and global DNA methylation observed in our case is worthy of further studies.

Our findings can shed also light on various factors associated with the environmental or biogeographic provenance. Alonso et al*.*^23^ propose a general rule that species with wider geographic ranges tend to demonstrate lower levels of DNA methylation. In our case, the opposite was true: *F. tatrae*, the narrow endemics, showed a lower level of DNA methylation. However, it is possible that grasses may be the exception to this rule, especially considering the special nature of their epigenome in comparison to eudicots^42^; indeed, only four of the 279 taxa analyzed by Alonso et al*.*^23^ were grass species.

It has been proposed that populations from different regions and different habitat conditions develop specific methylation patterns; in theory, such patterns help plants optimally match their reaction to the conditions in which they live^22^. Variations in DNA methylation between habitats have been reported in several studies^26,43,44^. Epiloci related to eco-environmental variables, particularly water availability and temperature, have been described for the allotetraploid complex from *Dactylorhiza*^45^, while DNA methylation differences were reported between vineyards growing in different sub-regions^46^. Our findings suggest that the level of DNA methylation occurring during stress may be significantly influenced by the general climatic provenience of specimens. Furthermore, the greatest influence appeared to be exerted by the general climate parameters in the original location, such as temperature extremes and the range between them, as well as the temperatures characterizing the driest or wettest parts of the year.

In our experiment, the differences observed between plants originating from different regions may be attributed to weather differences between seasons: in 2019, the weather conditions were more stressful than in 2018, and methylation levels appeared to be less dependent on the environmental provenance of individuals. Therefore, the effect of original local adaptation on DNA methylation level in such experiments appears to depend on level and duration of stress. Similar conclusions were stated by Richards et al*.*^44^, who report that while methylation patterns appear to be partly persistent (induced by original habitat and then maintained), the influence of the bioclimatic parameters of the original locations of the plants are modified by additional elements^44^. It should be noted that most of the regression models calculated in our study poorly describe the level of DNA methylation. It therefore appears that the analyzed variables modify the methylation level rather than independently shaping it.

## Materials and methods

### Common garden experiment and plant material sampling

Specimens of *F. amethystina* and *F. tatrae* were collected during field studies (Supplementary Table S1). These were grown in common garden experimental plots in the Botanical Garden in Lodz, Central Poland (51°45′12.5″N, 19°24′30.2″E). They had been grown together for at least a year before sampling. The experiment was conducted during two growing seasons, i.e. in the years 2018 and 2019. Each year, plant material was taken for total DNA methylation analysis during two periods of different weather conditions: (1) under water and temperature stress and (2) under control (favorable) conditions. The plant material was sampled at the time when plants were fully flowering. In 2018, stress condition samples were taken on 2nd June and controls on 2nd July; in 2019 they were taken on 17th June (stress conditions) and on 12th July (control). Plant material was taken from exactly the same plants during the control and stress conditions, and from precisely the same plants each studied year.

Sampling was performed according to good practice, described by Herrera and Bazaga^47^, with the consideration that variations in methylation level between different organs or developmental stages can differ between plant species^48^. All samples were obtained from the same organ and collected at identical developmental stages: i.e. fully-grown leaves from the middle part of fescue tufts. The sampled leaves were put into zipped plastic bags and frozen (< − 23 °C). Where it was possible, samples were taken from two or three specimens from the same population (biological replicates, Supplementary Table S1). Technical replicates (two per measurement) were performed for all samples during the ELISA test.

Each year, a few days after sampling the material for DNA methylation in control conditions, material was collected for phenotypic measurements.

Plant experiments were performed in accordance with relevant guidelines and regulations; plant material was collected with respective permission if necessary.

### Weather conditions during experiment

The day of sampling was chosen according to the ongoing analysis of weather conditions monitored by the meteorological station of the Institute of Meteorology and Water Management (IMGW), located in a similar landscape, 3.1 km away from the experimental plots.

Climatic data was obtained from the Accredited Station of the Institute of Meteorology and Water Management, National Research Institute (pol.: IMGW)—Lodz Lublinek. Daily and hourly data was obtained from the IMGW archives (IMGW website). The following data was used: air humidity (%, accuracy 0.1%), air temperature (°C, accuracy 0.1 °C), humidity deficiency (%, accuracy 0.1%), insolation (hours, accuracy 0.1 h/h). The parameters were calculated for seven days and 24 h prior to collection (Supplementary Fig. S1).

### Assessment and analysis of global DNA methylation

DNA was isolated from the frozen leaves of *F. amethystina* and *F. tatrae* using syngen Plant DNA MINI Kit following the manufacturer’s instructions. The concentration of the obtained DNA was measured with BioDrop DUO (biodrop). Samples were diluted using TE buffer to bring all to the same concentration (15 ng/μl). The DNA concentration was measured again to make sure it was equal, and any possible dilution adjustments were made. The global DNA methylation (%) (Supplementary Tables S2–S5) was detected by ELISA test using a MethylFlash Methylated DNA Quantification Kit (Colorimetric) (epigentek, USA), according to the manufacturer’s instructions. Signal Detection was performed using a Model 550 Microplate Reader (bio-rad).

Absolute differences in global DNA methylation level between stress and control conditions and variations were calculated in a given year: one calculation for all studied specimens and another for each species and cytotype. The results were analyzed using ‘lollipop plots with baseline’ in R packages^49^: ‘ggplot’ and ‘Ggally’^50^, and boxplots in Statistica v. 13.3^51^.

The differences in the global DNA methylation level between species, and cytotypes, in a given year, and between sampling conditions (i.e. stress vs. control) were assessed using non-parametric Kruskal–Wallis ANOVA (the data was not normally distributed) in Statistica v. 13.3^51^.

Correlation matrices for the obtained global DNA methylation levels during stress and control conditions in a given year were evaluated separately for each species and cytotype. Pearson’s correlation coefficients and their statistical significance were calculated, and correlation matrices were created, using R packages^49^: ‘psych’ and ‘corrplot’.

### Factors affecting global DNA methylation

#### Ploidy level of specimens

Based on earlier data^14,34^, there is no evidence that the *F. tatrae* were anything other than diploid. For *F. amethystina*, the ploidy level (diploid or tetraploid) of the individuals growing in the same experimental plots was tested using flow cytometry, in accordance with Rewicz et al*.*^25^ (Supplementary Table S1).

#### Phenotypic characteristics of specimens

In this study, the following continuous traits were chosen as signs of biological fitness based on previous studies: height of stalks, number of stalks and the number of spikelets on the stalk^52^.

As in the above-mentioned analyses, the material was representative of the species and cytotype: all specimens researched epigenetically were also analyzed morphologically. Comparability of the results was ensured by the fact that the plants were cultivated in the same monitored and controlled environment. From the studied specimens, all stalks were counted. The height and spikelet numbers were measured for five stalks per specimen (Supplementary Tables S2–S5).

#### Moisture level of the upper horizon of soil during sampling

Measurements of soil moisture level were taken during sampling, directly near the clumps of specimens, up to 5–10 cm below the ground (Supplementary Tables S2–S5). Values were recorded as % humidity with an accuracy of 0.1% using an SM150 Soil Moisture Kit (delta-t).

#### Geographic provenance of specimens

##### Altitude

The altitude of original locations of plants was determined using Google Earth. In addition, a script was developed for assigning the altitude to coordinates of the central points of the population locations (Supplementary Tables S1 and S2–S5).

#### Bioclimates in original locations

Thirty arc (~ 1 km) resolution raster data was used, incorporating 19 bioclimatic variables from the WorldClim database^53^. Nineteen bioclimatic variables were assigned to each location from the rasters (Supplementary Tables S2–S5). The values were extracted according to the coordinates of locations in the ArcGIS Desktop 9.2: Spatial Analyst tools, Extract values to point tool^54^.

### Identification of the most important quantitative factors according to stepwise regression

Stepwise regression was performed to identify the most important variables. The procedure consisted of iteratively removing predictors in the predictive model, so as to find the subset of variables resulting in the best model. In other words, all predictors (variables from the data set) were initially used in the procedure and then the least contributive predictors were iteratively removed: for the next iteration, the model that yielded the lowest AIC was retained. The procedure stop when removing the next one variable would deteriorate the quality of the model.

For each analyzed period (viz*.* the stress and control periods for 2018 and 2019—Supplementary Tables S2–S5), the analysis was conducted for the three sets of predictors: Model set (1) soil moisture + phenotypic data + chromosome number; Model set (2) altitude + bioclimatic variables of plant original locations, while Model set (3) included the most important variables from Model set 1 and Model set 2. The limitation of the procedure is that the variables should not be collinear. To identify collinearity among explanatory variables, variance inflation factors (VIF) were used. In our case, a large number of climatic variables were collinear; therefore, we decided not to remove a number of variables from the analysis, but to create data subsets without collinear variables in particular subsets. Following this, a stepwise regression procedure was run in each data subset. In the case of Model set 2, 18 such data subsets were created: each variable appeared in four data subsets, each time in a different variable configuration (18 separate models were used to check whether any of the variables may have an impact on the analyzed phenomenon). The list of variables that were found to be statistically significant are presented in Supplementary Tables S2–S5. The calculations were conducted by the ‘car’ package and base functions in R^49,54^.

## Supplementary Information

Supplementary Information.

## Data Availability

All data generated or analyzed during this study are included in this published article (and its Supplementary Information files).

## References

[1] Kelly AE, Goulden ML (2008). Rapid shifts in plant distribution with recent climate change. Proc. Natl. Acad. Sci. U.S.A..

[2] Wiens JJ (2016). Climate-related local extinctions are already widespread among plant and animal species. PLoS Biol..

[3] Swinnen J, Burkitbayeva S, Schierhorn F, Prishchepov AV, Müller D (2017). Production potential in the “bread baskets” of Eastern Europe and Central Asia. Global Food Secur..

[4] Henry RJ (2020). Innovations in plant genetics adapting agriculture to climate change. Curr. Opin. Plant Biol..

[5] Stokes C, Howden M (2010). Adapting Agriculture to Climate Change: Preparing Australian Agriculture, Forestry and Fisheries for the Future.

[6] Bräutigam K (2013). Epigenetic regulation of adaptive responses of forest tree species to the environment. Ecol. Evol..

[7] Yaish MW, Colasanti J, Rothstein SJ (2011). The role of epigenetic processes in controlling flowering time in plants exposed to stress. J. Exp. Bot..

[8] Yaish MW, Rout GR, Das AB (2013). DNA methylation-associated epigenetic changes in stress tolerance of plants. Molecular Stress Physiology of Plants.

[9] Suji KK, Joel AJ (2010). An epigenetic change in rice cultivars underwater stress conditions. Electron. J. Plant Breed..

[10] Peng H, Zhang J (2009). Plant genomic DNA methylation in response to stresses: Potential applications and challenges in plant breeding. Prog. Nat. Sci..

[11] Baduel P, Colot V (2021). The epiallelic potential of transposable elements and its evolutionary significance in plants. Philos. Trans. R. Soc. B.

[12] Labra M (2002). Analysis of cytosine methylation pattern in response to water deficit in pea root tips. Plant Biol..

[13] Wang W-S (2011). Drought-induced site-specific DNA methylation and its association with drought tolerance in rice (*Oryza sativa* L.). J. Exp. Bot..

[14] Šmarda P, Bureš P, Horová L, Foggi B, Rossi G (2008). Genome size and GC content evolution of *Festuca*: Ancestral expansion and subsequent reduction. Ann. Bot..

[15] Tomczyk PP, Kiedrzyński M, Jedrzejczyk I, Rewers M, Wasowicz P (2020). The transferability of microsatellite loci from a homoploid to a polyploid hybrid complex: An example from fine-leaved *Festuca* species (Poaceae). PeerJ.

[16] Piękoś-Mirkowa H, Mirek Z (2009). Distribution patterns and habitats of endemic vascular plants in the Polish Carpathians. Acta Soc. Bot. Pol..

[17] Kiedrzyński M, Zielińska KM, Rewicz A, Kiedrzyńska E (2017). Habitat and spatial thinning improve the Maxent models performed with incomplete data. J. Geophys. Res. Biogeosci..

[18] Rewicz A (2018). Morphometric traits in the fine-leaved fescues depend on ploidy level: The case of *Festuca*
*amethystina* L. PeerJ.

[19] Kiedrzyński M (2021). Tetraploids expanded beyond the mountain niche of their diploid ancestors in the mixed-ploidy grass *Festuca amethystina* L. Sci. Rep..

[20] Mounger J (2021). Epigenetics and the success of invasive plants. Philos. Trans. R. Soc. B.

[21] Bewick AJ, Schmitz RJ (2015). Epigenetics in the wild. Elife.

[22] Sahu PP (2013). Epigenetic mechanisms of plant stress responses and adaptation. Plant Cell Rep..

[23] Alonso C (2019). Interspecific variation across angiosperms in global DNA methylation: Phylogeny, ecology and plant features in tropical and Mediterranean communities. New Phytol..

[24] Angers B, Castonguay E, Massicotte R (2010). Environmentally induced phenotypes and DNA methylation: How to deal with unpredictable conditions until the next generation and after. Mol. Ecol..

[25] Batog J, Wawro A (2019). Process of obtaining bioethanol from sorghum biomass using genome shuffling. Cellul. Chem. Technol..

[26] Richards CL, Schrey AW, Pigliucci M (2012). Invasion of diverse habitats by few Japanese knotweed genotypes is correlated with epigenetic differentiation. Ecol. Lett..

[27] Li N (2019). DNA methylation repatterning accompanying hybridization, whole genome doubling and homoeolog exchange in nascent segmental rice allotetraploids. New Phytol..

[28] Róis AS (2013). Epigenetic rather than genetic factors may explain phenotypic divergence between coastal populations of diploid and tetraploid *Limonium* spp. (Plumbaginaceae) in Portugal. BMC Plant Biol..

[29] Li A (2011). DNA methylation in genomes of several annual herbaceous and woody perennial plants of varying ploidy as detected by MSAP. Plant Mol. Biol. Rep..

[30] Sokolova DA, Vengzhen GS, Kravets AP (2013). An Analysis of the correlation between the changes in satellite DNA methylation patterns and plant cell responses to the stress. Cell Bio.

[31] Johnson LI, Tricker PJ (2010). Epigenomic plasticity within populations: Its evolutionary significance and potential. Heredity.

[32] Zheng X (2013). Transgenerational variations in DNA methylation induced by drought stress in two rice varieties with distinguished difference to drought resistance. PLoS One.

[33] Karan R, DeLeon T, Biradar H, Subudhi PK (2012). Salt Stress induced variation in DNA methylation pattern and its influence on gene expression in contrasting rice genotypes. PLoS One.

[34] Richards CL, Pigliucci M (2020). Epigenetic inheritance. A decade into the extended evolutionary synthesis. Paradigmi.

[35] Chelaifa H, Monnier A, Ainouche M (2010). Transcriptomic changes following recent natural hybridization and allopolyploidy in the salt marsh species *Spartina* × *townsendii* and *Spartina*
*anglica* (Poaceae). New Phytol..

[36] Al-Lawati A, Al-Bahry S, Victor R, Al-Lawati AH, Yaish MW (2016). Salt stress alters DNA methylation levels in alfalfa (*Medicago* spp.). Genet. Mol. Res..

[37] Lewandowska-Gnatowska E (2014). Is DNA methylation modulated by wounding-induced oxidative burst in maize?. Plant Physiol. Biochem..

[38] Marfil C (2019). Changes in grapevine DNA methylation and polyphenols content induced by solar ultraviolet-B radiation, water deficit and abscisic acid spray treatments. Plant Physiol. Biochem..

[39] Zedek F (2020). Endopolyploidy is a common response to UV-B stress in natural plant populations, but its magnitude may be affected by chromosome type. Ann. Bot..

[40] Pandey N, Pandey-Rai S (2015). Deciphering UV-B-induced variation in DNA methylation pattern and its influence on regulation of DBR2 expression in *Artemisia*
*annua* L. Planta.

[41] Molinier J (2017). Genome and epigenome surveillance processes underlying UV exposure in plants. Genes.

[42] Niederhuth CE (2016). Widespread natural variation of DNA methylation within angiosperms. Genome Biol..

[43] Lira-Medeiros CF (2010). Epigenetic variation in mangrove plants occurring in contrasting natural environment. PLoS One.

[44] Richards CL, Verhoeven KJF, Bossdorf O, Wendel JF, Greilhuber J, Dolezel J, Leitch IJ (2012). Evolutionary significance of epigenetic variation. Plant Genome Diversity.

[45] Paun O (2010). Stable epigenetic effects impact adaptation in allopolyploid orchids (Dactylorhiza: Orchidaceae). Mol. Biol. Evol..

[46] Xie H (2017). Global DNA methylation patterns can play a role in defining terroir in grapevine (*Vitis*
*vinifera* cv. Shiraz). Front. Plant Sci..

[47] Herrera CM, Bazaga P (2010). Epigenetic differentiation and relationship to adaptive genetic divergence in discrete populations of the violet *Viola*
*cazorlensis*. New Phytol..

[48] Portis E, Acquadro A, Comino C, Lanteri S (2004). Analysis of DNA methylation during germination of pepper (*Capsicum*
*annuum* L.) seeds using methylation-sensitive amplification polymorphism (MSAP). Plant Sci..

[49] R Core Team. R: A language and environment for statistical computing. http://www.R-project.org (R Foundation for Statistical Computing, 2013).

[50] Schloerke, B. *et al*. GGally: Extension to “ggplot2” R package version 2.1.0. https://CRAN.R-project.org/package=GGally (2021).

[51] StatSoft, Inc. STATISTICA (Data Analysis Software System), Version 10. http://www.statsoft.com (2011).

[52] Tomczyk P (2019). Phenotypic measurement of inbreeding depression in grasses—An overview of traits (Fenotypowe miary depresji wsobnej u traw—przegląd cech). Wiad. Bot..

[53] Fick SE, Hijmans RJ (2017). WorldClim 2: new 1km spatial resolution climate surfaces for global land areas. Int. J. Climatol..

[54] Fox J, Weisberg S (2019). An {R} Companion to Applied Regression.

